# Effect of corneal refractive surgery on accommodative and binocular dysfunctions among civilian pilots in Southwest China

**DOI:** 10.1186/s12886-021-01855-0

**Published:** 2021-02-19

**Authors:** Ye Wu, Zhen Zhang, Meng Liao, Qi Li, Xue Lin Tang, Longqian Liu

**Affiliations:** 1 Department of Ophthalmology, West China Hospital, Sichuan University, Chengdu, 37 Guoxue Xiang, Chengdu, Sichuan Province 610041 P. R. China; 2 Department of Ophthalmology, Chengdu Civil Aviation Medical Center, Chengdu, Sichuan Province P. R. China; 3 Department of Internal, Chengdu Civil Aviation Medical Center, Chengdu, Sichuan Province P. R. China

**Keywords:** Accommodative dysfunctions, Binocular dysfunctions, Refractive surgery, Civilian pilot

## Abstract

**Background:**

To analyze whether corneal refractive surgery (CRS) is associated with the distribution of different accommodative dysfunctions (ADs) and binocular dysfunctions (BDs) in civilian pilots. A further aim was to analyze the percentages and visual symptoms associated with ADs and/or BDs in this population.

**Methods:**

One hundred and eight civilian pilots who underwent CRS from January 2001 to July 2012 (age: 30.33 ± 4.60 years) were enrolled, the mean preoperative SE was − 1.51 ± 1.15 D (range: − 1.00- − 5.00 D). Ninety-nine emmetropic civilian pilots (age: 29.64 ± 3.77 years) who were age- and sex-matched to the CRS group were also enrolled. Refractive status, accommodative and binocular tests of each subject were performed. Visually related symptoms were quantified using the 19-item College of Optometrists in Vision Development Quality of Life (COVD-QOL) questionnaire. The 19 items were summed to obtain visual symptom scores that might indicate visual dysfunctions. The chi-square test was used to analyze differences in percentages of ADs and/or BDs between the CRS and emmetropic groups. The Mann-Whitney U test was used to compare visual symptom scores between pilots with ADs and/or BDs and pilots with normal binocular vision.

**Results:**

No significant difference was observed between the CRS and emmetropic groups in the overall prevalence of ADs and BDs (15.7% and 15.2% in the CRS and emmetropic groups, respectively; *P* = 0.185). ADs were present in 4.63% and 3.03% of the CRS and emmetropic group, respectively. BDs were observed in 11.1% and 12.1% of the CRS and emmetropic group, respectively, yielding no significant differences between the groups in the prevalence of ADs or BDs (AD: *P* = 0.094; BD: *P* = 0.105). Pilots with ADs and/or BDs had significantly more visual symptoms than pilots with normal binocular vision (*p* < 0.001).

**Conclusions:**

CRS for civilian pilots with low-moderate myopia might not impact binocular functions. ADs and/or BDs commonly occur in both emmetropia pilots and pilots who undergo CRS, and pilots with ADs and/or BDs are associated with increased symptoms. This study confirms the importance of a full assessment of binocular visual functions in detecting and remedying these dysfunctions in this specific population.

**Supplementary Information:**

The online version contains supplementary material available at 10.1186/s12886-021-01855-0.

## Background

The high prevalence of myopia in China [1, 2] has been a long-standing problem for the Civil Aviation Administration of China (CAAC), as the need to maintain stringent aeromedical visual standards must be balanced with the conflicting requirement of meeting aircrew recruitment demands. To increase the recruitment pool of potential civilian pilots, the CAAC ultimately approved corneal refractive surgery (CRS) for civilian pilots in 2006 [3]. Previous studies have reported the decompensation of binocular vision anomalies after CRS in clinical populations, such as postoperative strabismus and diplopia [4, 5]. However, the impact that CRS may have on binocular visual functions among pilots has not been reported.

ADs and/or BDs are visual disorders that affect binocular vision and visual performance. Early detection of clinically significant ADs and/or BDs is important, as some of these deviations may decompensate and become strabismic, resulting in the loss of stereopsis, suppression and a wide variety of associated visual and mental symptoms [6–10]. Most of these studies are performed in pediatric populations [11–14], high school [15] and university students [16, 17]. To our knowledge, reports are lacking on the frequency of ADs and/or BDs among pilots. This population is of great interest because their workforce has perhaps the greatest amount of near work of any population [18], and the presence of ADs and/or BDs may result in visual symptoms that affect flight performance and leisure activities. Therefore, this matter should be investigated, as flying is widely viewed as a mentally and visually demanding task within a degraded visual environment.

Accordingly, the main purpose of this study is to determine whether CRS is associated with the distribution of different ADs and/or BDs in civilian pilots. A further aim was to analyze the percentages of ADs and/or BDs in this population, and to decide whether visual symptoms are more common in pilots with ADs and/or BDs.

## Methods

### Subjects

This prospective study is part of a large study intended to analyze the visual performances of civilian pilots after CRS. This study investigated the prevalence of ADs and/or BDs among pilots who underwent CRS and emmetropic pilots, and it was conducted between October 2018 and April 2019. This study was approved by the local ethics committee [No 2014 (33), 1-6-2015], and written informed consent was obtained from all subjects.

The enrolled participants were grouped into CRS and emmetropic groups based on the spherical equivalent (SE) of noncycloplegic refraction and CRS history. The inclusion criteria for civilian pilots who underwent CRS were as follows: (1) aged 18–35 years; (2) unremarkable general and ocular health; and (3) best-corrected visual acuity of at least 20/20 Snellen visual acuity. The inclusion criteria for emmetropic civilian pilots included aged 18–35 years and emmetropic (both eyes with SE between + 0.50 diopters (D) and − 0.50 D). The exclusion criteria for both groups were the presence of strabismus, a history of strabismus surgery or intraocular surgery, anisometropia greater than 1.5 D, absence of binocular vision, and anterior segment pathologic conditions.

The two groups were given a questionnaire assessment, refractive examination, and accommodative and binocular tests.

The procedures included the following:

### Questionnaire

The questionnaire used in this study was adapted from the 19-item College of Optometrists in Vision Development (COVD) Quality of Life (QOL) assessment, which has good test-retest reliability in measuring subjects’ visual symptoms in general [19, 20]. It was translated into Chinese based on the Brislin translation model [21]. Participants were instructed to rate the presence of any symptom on a five-point Likert scale. Each item had five possible answers with an associated value — always (4), frequently (3), occasional (2), seldom (1), and never (0). A total score of 20 and above is a concern and further evaluation is needed.

### Refractive examination

The refractive examination was performed using static retinoscopy and subjective refraction (RT-600, Nidek Co. Ltd.). The subjective refraction was determined using a monocular fogging method with a cross-cylinder followed by binocular balancing to a standard endpoint of maximum plus for best visual acuity. If a subject was found to have a significant refractive error (myopia ≤ − 0.50 D, hyperopia ≥ + 0.50 D, astigmatism≥0.50 D) or if a change in refractive error of more than 0.50 D was detected in the spherical or cylindrical component during the refraction, spectacles were prescribed and accommodative and binocular tests were then performed 2 weeks after wearing the spectacle prescription.

### Accommodative and binocular tests

The tests involved assessment of the direction and magnitude of the distance and near horizontal and vertical phoria with a cover test and prism bar. Accommodative convergence/accommodation ratios were measured with both calculated and gradient methods. Positive and negative fusional vergence were measured at far and near distance with Risley prisms (Nidek Co. Ltd.) using an accommodative target of 20/30 visual acuity at 40 cm. Positive and negative relative accommodation was measured with plus and minus lenses, respectively, using an accommodative target of 20/30 visual acuity at 40 cm until a sustained blur was perceived. The near point of convergence was measured with an accommodative target of 20/30 visual acuity at 40 cm and moving the target away from the subject at a speed of approximately 1 to 2 cm per second until the break and recovery findings. Binocular accommodative facility was measured at 40 cm using ±2.00 D flipper lenses and the 20/30 letter line on the Vectogram 9 (Tianjin Oput Visual Training Co. Ltd.), which included suppression control for the binocular measurement. Monocular accommodative facility was measured by the same method but without polarized glasses and with the nonviewing eye occluded. Vergence facility was measured using a 12Δ base-out/3Δ base-in prism flipper at 40 cm. The subjects were instructed to report clarity as soon as the letters were clear and single. Dynamic retinoscopy with the monocular estimate method at 40 cm was performed with the result of the subjective refraction placed in a trial frame while using trial lenses. Monocular accommodative amplitude was measured by the push-up method with a single 20/30 Snellen line target in free space.

### Diagnosis of dysfunctions

This study decided to classify dysfunction based on the number of clinical signs associated with each dysfunction according to other studies (Table 1) [6, 10, 11, 22]. Consequently, the subjects who had any type of visual symptom and clinical signs were classified as symptomatic subjects and then included in the prevalence study. The subjects without visual symptoms had normal clinical findings, and they were classified as normal binocular vision (NBV). The subjects who had abnormal clinical findings but no visual symptoms were classified as asymptomatic subjects.

**Table 1:** Diagnostic criteria for non-strabismic binocular and accommodative dysfunctions

Convergence Insufficiency	Requires 1, 2, and 3
	1. Near exophoria at least 4△ greater than distance exophoria 2. NPC break point ≥6 cm 3. Reduced near PFV (break point ≤15△ or failed Sheard’s criterion)
Convergence Excess	Requires 1 and at least 1 sign from 2 ~ 3
	1. Near esophoria greater than distance esophoria by ≥4△ 2. Reduced near NFV, ≤8/16/7 for blur, break and recovery (at least one of three) 3. Near VF ≤12 cpm
Divergence Insufficiency	Requires 1 or 2 + 3
	1. Distance esophoria greater than near esophoria by ≥10△ 2. Distance esophoria greater than near esophoria by ≥4△ 3. Reduced distance NFV (break point ≤4△ or failed Sheard’s criterion)
Divergence Excess	Requires 1 or 2 + 3
	1. Distance exophoria greater than near esophoria by ≥10△ 2. Distance exophoria greater than near esophoria by ≥4△ 3. Reduced distance PFV, ≤ 4/ 10/ 5 Δ (at least one of three)
Basic Exophoria	Requires 1, 2 and at least 1 sign from 3 ~ 5
	1. Difference between near and distance exophoria ≤3△ 2. Subjects needs to be exophoria at both distant and near 3. PFV at far ≤4 /10/ 5Δ and ≤ 11/14/3 Δ at near (at least one of three) 4. NPC break point ≥6 cm 5. Near VF ≤12 cpm
Fusional Vergence Dysfunction	Requires 1, 2 and at least 1 sign from 3 ~ 4
	1. No significant phoria at distance and near (distance: exophoria ≤2△ to orthophoria; near: exophoria ≤5△ to orthophoria) 2. No other vergence dysfunction diagnosed 3. Reduced NFV or PFV (PFV break point ≤15△ or NFV break point≤7△ or failed Sheard’s criterion) 4. Near VF ≤12 cpm
Accommodative Insufficiency	Requires 1
	1.Monocular AA at least 2 D below minimum age-based norms as defined by Hofstetter’s formula (15-age/4)
Accommodative Infacility	**(**Requires 1 or 2
	1.MAF ≤ 6 cpm with ±2.00 D lenses 2.BAF ≤ 3 cpm with ±2.00 D lenses
Accommodative excess	1. Variable visual acuity findings 2. Variable static retinoscopy and subjective refraction 3. MAF ≤ 6 cpm with + 2.00 D lenses 4. BAF ≤ 3 cpm with + 2.00 D lenses 5. MEM < + 0.25 D 6. NRA < + 1.50 D

*NPC* Near point of convergence, *PFV* Positive fusional vergence, *NFV* Negative fusional vergence, cpm cycle per minute, *AA* Amplitude of accommodation, *BAF* Binocular accommodative facility, *MAF* Monocular accommodative facility, *MEM* Monocular estimated method, *NRA* Negative relative accommodation, *VF* Vergence facility

### Data analysis

All statistical analyses were performed using SPSS 18.0 (SPSS Inc., Chicago, Illinois, USA) for Windows. Descriptive statistics were calculated for ADs and/or BDs, and the chi-square test was used to analyze differences in frequencies and percentages between the CRS and emmetropic groups. The Mann-Whitney U test was performed to compare clinical findings between the CRS and emmetropic groups. Cronbach’s alpha coefficient and a principal component factor analysis provided data on the reliability and validity of the COVD-QOL questionnaire, respectively. The Mann-Whitney U test was used to compare visual scores between pilots with ADs and/or BDs and NBV subjects. The Mann-Whitney U test was also used to compare clinical findings between symptomatic CI and asymptomatic CI. A *P* value of < 0.05 was considered significant.

## Results

### Demographic data

Only 110 civilian pilots had undergone CRS surgery in Southwest China; two were excluded because they were older than 35 years. Therefore, 108 civilian pilots who underwent CRS from January 2001 to July 2012 were included. The mean preoperative SE was − 1.51 ± 1.15 D (range: − 1.00- − 5.00 D). The mean postoperative SE was − 0.023 ± 0.52 D. A total of 147 eyes (68.1%) were within ±0.50 D of emmetropia.

This study also enrolled 104 emmetropic civilian pilots. Of those, 5 were excluded from the analyses due to the presence of refractive errors (*n* = 4) and anisometropia (*n* = 1). The final sample included 99 emmetropic pilots who were age- and sex-matched to the CRS group. No significant difference was observed for SE between the CRS and emmetropic groups (*p* = 0.268) (Table 2).

**Table 2:** Demographic, percentages and clinical findings of the subjects in both groups

	CRS group	Emmetropia group	*P* value
Age (years):	30.33 ± 4.60 (23–35)	29.64 ± 3.77 (23–35)	0.296
Gender (M/F):	106/2	98/1	0.533
Spherical equivalent (D):	−0.023 ± 0.52	0.023 ± 0.30	0.268
Diagnosis:			
ADs and BDs	17 (15.7%)	15 (15.2)	0.524
Asymptomatic CI	8 (7.41%)	7 (7.07%)	0.570
NBV subjects	83 (76.85%)	77 (77.78%)	–
Total	108 (100%)	99 (100%)	–
Clinical findings			
AA (right eye only, D)	9.65 ± 2.06	9.27 ± 1.78	0.206
MAF (right eye only, cpm)	12.81 ± 6.93	11.80 ± 6.64	0.434
BAF (cpm)	12.41 ± 6.44	10.73 ± 5.79	0.068
MEM (right eye only, D)	−0.076 ± 0.55	−0.025 ± 0.50	0.494
PRA (D)	−2.66 ± 1.49	−2.35 ± 1.37	0.122
NRA (D)	1.87 ± 0.60	1.73 ± 0.60	0.136
Phoria (Δ)			
Near	−3.73 ± 4.94	−3.59 ± 5.32	0.505
Distance	−1.56 ± 2.51	−1.53 ± 2.63	0.481
PFV (Near break point) (Δ)	23.00 ± 7.72	22.37 ± 7.72	0.468
NFV (Near break point) (Δ)	20.53 ± 5.39	19.89 ± 4.81	0.246

*CRS* Corneal refractive surgery, *SE* Spherical equivalent, *ADs* Accommodative dysfunctions, *BDs* Binocular dysfunctions, *CI* Convergence insufficiency, *NBV* Normal visual binocular, *AA* Accommodative amplitude, *BAF* Binocular accommodative facility, *MAF* Monocular accommodative facility, *MEM* Monocular estimated method, *NPC* Near point of convergence, *PFV* Positive fusional vergence, *NFV* Negative fusional vergence, *PRA* Positive relative accommodation, *NRA* Negative relative accommodation

### Comparison of ADs and BDs

No significant difference was observed between the CRS and emmetropic groups for the overall prevalence of ADs and BDs (χ^2^ = 0.14, *p* = 0.524). ADs were present in 4.63% and 3.03% of the CRS and emmetropic group, respectively. BDs were observed in 11.1% and 12.1% of the CRS and emmetropic group, respectively. Convergence insufficiency (CI) was the most prevalent anomaly of binocular vision among all subtypes in both the CRS and emmetropic groups. Other subtypes showed a prevalence close to 1%; hence, statistical analyses were restricted to CI (Table 3).

**Table 3:** Prevalence of accommodative and binocular dysfunctions in the CRS and emmetropia civilian pilots

	CRS group		Emmetropia group		
Diagnosis	Number	%	Number	%	*P* value
Accommodative dysfunctions:	5	4.63	3	3.03	0.410
Accommodative excess	2	1.85	2	2.02	–
Accommodative insufficiency	2	1.85	0	0.00	–
Accommodative infacility	1	0.93	1	1.01	–
Binocular dysfunctions:	12	11.1	12	12.1	0.495
Convergence excess	2	1.85	2	2.02	–
Convergence insufficiency	5	4.63	6	6.06	0.440
Fusional vergence dysfunction	2	1.85	1	1.01	–
Divergence excess	2	1.85	1	1.01	–
Basic exophoria	1	0.93	1	1.01	–
Divergence insufficiency	0	0.00	1	1.01	–
Total	17	15.7	15	15.2	0.524

*CRS* Corneal refractive surgery

### Visual symptoms

Regarding the COVD-QOL questionnaire, the Cronbach’s alpha coefficient of the reliability analysis was 0.87, and the Kaiser-Meyer-Olkin value of validity analysis was 0.73, indicating that the questionnaire used in this study was valid. The mean total COVD-QOL score was 19.18 ± 9.55 for the CRS group and 18.90 ± 9.64 for the emmetropic group. No significant difference was found between CRS and emmetropia group for the total COVD-QOL score (U = 488.50, *p* = 0.771).

Both groups scored a higher percentage in the “never” category for 8 of the COVD-QOL items; hence, the statistical analyses were restricted to another 11 items: time use, copying a chalkboard, losing attention, skips, holding reading close, decreased comprehension, omissions, words running together, up/down, misaligns, and headaches. No significant differences were observed between the CRS and emmetropia group for these 11 items (all of them had a *p*>0.05).

**Fig. 1:**
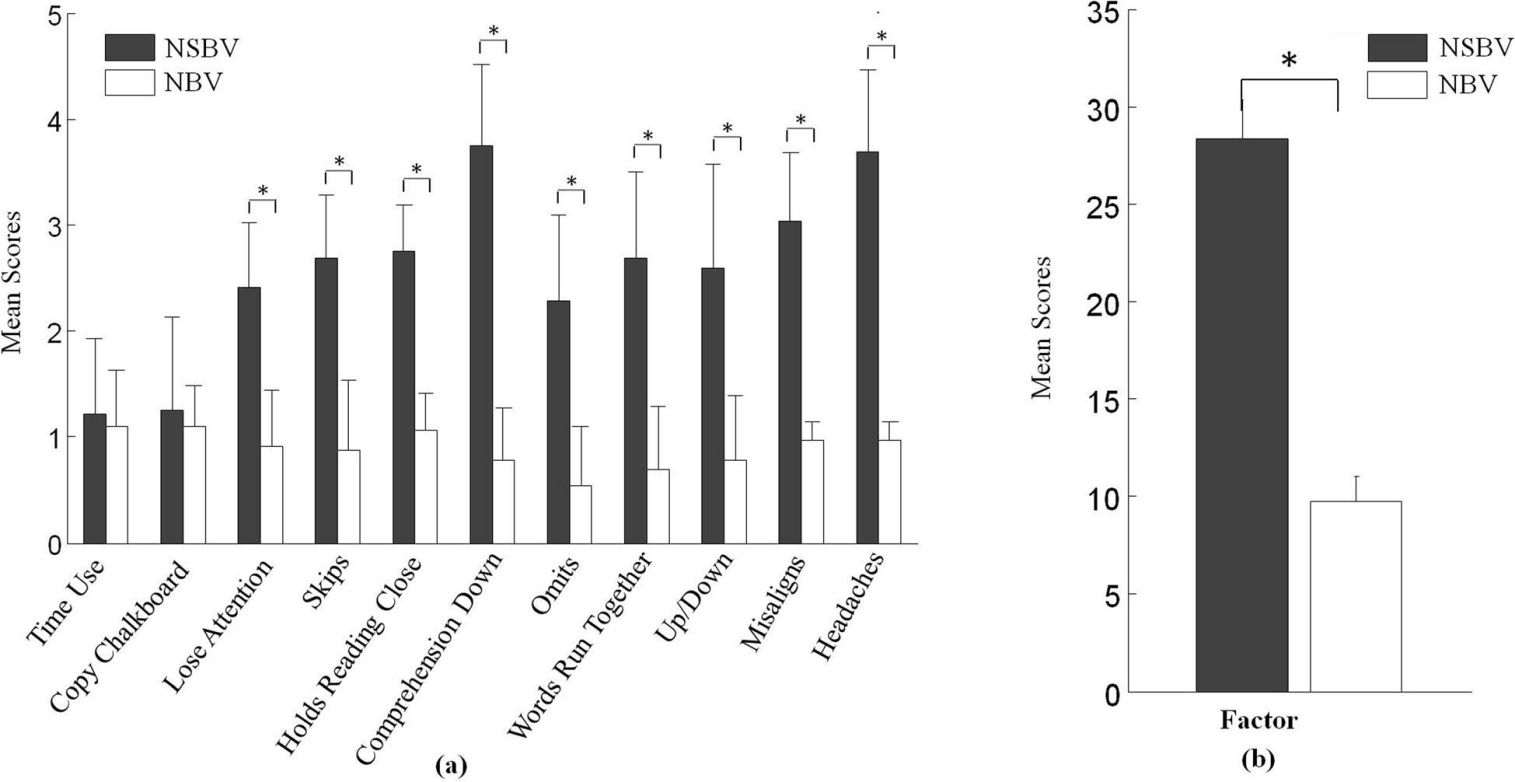
**a** Symptom means of NSBV and NBV subjects on the 11 items of the COVD-QOL checklist. **b** Symptom means of NSBV and NBV subjects on the mean total COVD-QOL score

The CRS and emmetropia group data were analyzed together to determine whether visual symptoms are more common in pilots with ADs and/or BDs (NSBVAs group) than pilots with normal binocular vision (NBV group), and the results revealed that the NSBVAs group had significant visual symptoms including losing attention (U = 41.50, *p* < 0.001), skips (U = 36.50, *p* < 0.001), holding reading close (U = 12.00, *p* < 0.001), Comprehension Down (U = 0.50, *p* < 0.001), omissions (U = 64.00, *p* < 0.001), words running together (U = 33.00, *p* < 0.001), up/down (U = 68.00, *p* < 0.001), misaligns (U = 0.00, *p* < 0.01), and headaches (U = 15.50, *p* < 0.001) and a significant mean total COVD-QOL score (U = 0.00, *p* < 0.001) (Fig. 1).

### Comparison of asymptomatic CI and symptomatic CI

Interestingly, only subjects with CI had abnormal clinical findings with no visual symptoms. Subjects with symptomatic CI had significantly lower accommodative amplitude (*p* = 0.005), binocular accommodative facility (*p* < 0.001) and monocular accommodative facility (p < 0.001) than asymptomatic CI subjects (Table 4).

**Table 4:** Comparison of accommodative and binocular findings between symptomatic and asymptomatic subjects with convergence insufficiency

Parameters	Symptomatic CI (*n* = 11) Mean ± SD	Asymptomatic CI (*n* = 15) Mean ± SD	*P* value
AA (D)	5.9 ± 2.1	7.8 ± 1.4	0.005^a^
MAF (right eye only, cpm)	5 ± 6	9 ± 5	<0.001^a^
BAF (cpm)	3 ± 3	6 ± 5	<0.001^a^
MEM (right eye only, D)	+ 0.15 ± 0.42	+ 0.11 ± 0.24	0.069
PRA (D)	−2.11 ± 0.23	−2.05 ± 0.57	0.174
NRA (D)	+ 1.41 ± 0.33	+ 1.21 ± 0.42	0.084
Phoria (Δ)			
Near	−13.1 ± 4.2	−11.7 ± 3.8	0.056
Distance	−2.1 ± 0.7	− 2.8 ± 1.6	0.125
PFV (Near break point) (Δ)	10.1 ± 7.4	8.8 ± 6.9	0.055
NFV (Near break point) (Δ)	11.3 ± 4.4	11.0 ± 3.1	0.154

*AA* Accommodative amplitude, *BAF* Binocular accommodative facility, *MAF* Monocular accommodative facility, *MEM* Monocular estimated method, *NPC* Near point of convergence, *PFV* Positive fusional vergence, *NFV* Negative fusional vergence, *PRA* Positive relative accommodation, *NRA* Negative relative accommodation, ^a^ Statistically significant

## Discussion

The present study demonstrated no significant differences in the prevalence of ADs and/or BDs between the CRS and emmetropic pilots, and binocular vision dysfunction was a common finding in this population. To our knowledge, this was the first study to investigate ADs and/or BDs among civilian pilots. Thus, some aspects of these abnormalities need to be addressed.

As shown in Table 3, the lack of significant differences in the prevalence of ADs and/or BDs between the CRS and emmetropic groups suggests that CRS in civilian pilots with low-moderate myopia might not generally impact binocular functions. These findings are consistent with the results of a recent study [23], which showed that diplopia and strabismus are rare complications after CRS in the U.S. military population. According to Kushner et al. [24], patients with less than 4 D of anisometropia, no prisms in their spectacles, and no history of diplopia or strabismus should be considered to have low risk of postoperative binocular function decompensation. In addition, García-Montero M et al. [4] found that most decompensation of binocular vision after CRS were in fact preoperative disorders. In this study, the mean preoperative SE of the CRS group was − 1.51 ± 1.15 D with no other significant preoperative medical histories. Thus, the pilots who underwent CRS met the low risk standards of postoperative ADs and/or BDs. Therefore, it might be reasonable that the differences in the prevalence of ADs and/or BDs between CRS and emmetropic groups were nonsignificant in this study.

In this study, the prevalence of overall ADs and/or BDs was estimated to be 15.7 and 15.2% in the CRS and emmetropia pilots, respectively. This study obtained a lower prevalence of ADs and/or BDs than those reported in three studies [16, 25, 26] employing adult population. Lara et al. [25] examined a sample of 265 symptomatic participants who consecutively attended an optometric clinic and found that 59 (22.3%) presented some type of AD or BD. Martin et al. [26] examined the prevalence of ADs and/or BDs in a clinical population of 415 Chinese participants, finding that 178 patients (42.9%) in the total sample had general binocular disorders. The samples from both studies were taken from a clinical population seeking solutions to visual symptoms, which might have contributed to the higher prevalence of visual anomalies than the general population. In addition, Martin et al. [26] deliberately did not consider subjective symptoms when classifying participants with diagnostic criteria. Thus, the data obtained by Martin et al. [26] might have provided an overestimation of binocular vision dysfunctions.

Esteban [17] selected 65 university students aged approximately 22 years old, 32.3% of whom showed ADs or BDs. This percentage is much higher than those obtained in the present study. Although the participants included in these studies were all university students and thus similar in age to the subjects in this study, drawing comparisons between them is still difficult, as each study included different populations, measurement methods and diagnostic criteria [9, 10]. For example, Esteban [17] applied “moderate to high exophoria at near >6△” to diagnose CI, while the present study adopted the standard criteria (“Near exophoria at least 4△ greater than distance exophoria”) suggested by Scheiman [6, 22] to diagnose CI. Therefore, the diagnostic criteria of ADs and/or BDs used in these two studies differ substantially, and this should be considered one of the main factors leading to the varying prevalence figures between studies.

The prevalence in this study was slightly higher than that obtained by Ángel [16], who found that 23 university students (13.15%) among the total sample presented some type of AD and/or BD. They used criteria for diagnosing ADs and/or BDs similar to those used in the present study. However, the prevalence of ADs and/or BDs was different because of the characteristics of the study participants. The participants of the present study were civilian pilots who are required to perform considerable amounts of near work, such as reading a panel during a long-duration flight; thus, they are more likely to develop symptoms and signs related to ADs and/or BDs. In addition, lack of sleep [27], fatigue [28], and cervical symptoms [29] are also known to aggravate the problem [30]. Therefore, studying the prevalence of ADs and/or BDs among this specific population is important for planning appropriate intervention.

As shown in Fig. 1, regarding visual symptoms, the higher COVD-QOL scores in this study suggest that most pilots with ADs and/or BDs indeed experience many visual problems in their daily lives. These findings are consistent with the results of previous studies [20, 31], which showed that individuals with binocular vision anomalies had more visual discomfort symptoms than those with normal binocular vision. These visual complaints may include asthenopia, headache, blurred vision, loss of concentration when reading or doing near work [6–8]. These visual symptoms may have a negative effect on flight performance and leisure activities. Headaches, for example, can diminish pilots’ quality of life by giving them constant pain. Asthenopia and blurred vision can seriously affect pilots’ daily activities. Hence, all pilots who complain of visual symptoms should be tested for ADs and/or BDs. Detecting and managing these dysfunctions as early as possible are important, as pilots are required to operate under both physiologically and psychologically stressful conditions, and they often face a high visual workload demand within a degraded visual environment [18].

This study separately analyzed subjects with an asymptomatic binocular vision anomaly (Table 4), revealing that only subjects with CI had abnormal clinical measurements without symptoms. The results of this study are similar to the results reported by Hussaindeen et al. [11], who found that 58 school children (6.3%) in the total sample were asymptomatic but still failed the binocular vision tests.

Several factors may account for this mismatch between signs and symptoms. Firstly, some professionals have argued that CI is not a highly symptomatic condition. Some subjects with CI who were not symptomatic might have suppression, avoidance of near visual tasks, or monocular occlusion [32, 33], but this was not assessed directly in this study. Secondly, this study also revealed significantly lower accommodative amplitude and binocular accommodative facility in symptomatic subjects with CI compared with asymptomatic subjects with CI. According to the results of Marran et al. [32], children with accommodative insufficiency (AI) only and children with both AI and CI had more visual symptoms than children with CI only. The outcomes obtained in the current study further corroborate the conclusion of Marran et al. [32] that elevated symptoms in CI may be the result of comorbid AI. Therefore, determining the symptoms specific to CI due to the high comorbidity of CI and accommodative dysfunction would be beneficial for future studies.

Furthermore, the subjective responses of pilots may not be reliable. Most binocular tests used to diagnose ADs and/or BDs are based on subjective responses. However, this study cohort included two groups of motivated and highly competitive pilots who were required to faithfully complete all measurements, and they may have “overachieved” on the subjective response tests. Therefore, the prevalence results of ADs and/or BDs among the civilian pilots in this study represent the best-case scenario.

This study has several limitations. Firstly, LASIK, LASEK or PRK are different procedures, differences in the distribution of ADs and/or BDs should have been discussed. However, there were only 110 pilots had CRS in Southwest China; therefore, the small number of participants in the present study did not allow for meaningful further subanalyses of ADs and/or BDs. Secondly, although only adult participants were included, cycloplegia was not applied to avoid disrupting the evaluation of accommodation. However, the plus lens (+ 2.00 D) test was conducted on all participants to exclude latent hyperopia. Lastly, these data were based on the currently outdated broad beam laser and cannot be directly compared with contemporary techniques, such as femtosecond laser technology. Further studies are needed in this area.

## Conclusions

These findings suggest that CRS for civilian pilots with low-moderate myopia might not have a general impact on binocular function. ADs and/or BDs are commonly present in pilots with both CRS and emmetropia. Pilots with increased visual symptoms may benefit from binocular vision evaluation to assess for the presence of ADs and/or BDs, and future research investigating the potential risks associated with aviation accident experience of civilian pilots with ADs and/or BDs might provide more insight into improving their vision efficiency and daily lives.

## Supplementary Information

**Additional file 1: Appendix.** 19-item College of Optometrists in Vision Development Quality of Life (COVD-QOL) questionnaire.

## Data Availability

The datasets used and analyzed during the current study are available from the corresponding author on reasonable request.

## Abbreviations

CRS: Corneal refractive surgery
ADs: Accommodative dysfunctions
BDs: Binocular dysfunctions
CAAC: Civil Aviation Administration of China
SE: Spherical equivalent
D: Diopters
COVD: College of Optometrists in Vision Development
QOL: Quality of Life
NBV: Normal binocular vision
CI: Convergence insufficiency
AI: Accommodative insufficiency
NPC: Near point of convergence
PFV: Positive fusional vergence
NFV: Negative fusional vergence
cpm: Cycle per minute
AA: Amplitude of accommodation
BAF: Binocular accommodative facility
MAF: Monocular accommodative facility
MEM: Monocular estimated method
NRA: Negative relative accommodation
VF: Vergence facility
PRA: Positive relative accommodation
