# Supplementary material for: Effect of corneal refractive surgery on accommodative and binocular dysfunctions among civilian pilots in Southwest China
Source: BMC Ophthalmol. 2021 Feb 19;21:95. doi: 10.1186/s12886-021-01855-0 (PMC7893863; doi:10.1186/s12886-021-01855-0)
Supplement: Supplementary file 1 — Additional file 1: Appendix. 19-item College of Optometrists in Vision Development Quality of Life (COVD-QOL) questionnaire. [file 12886_2021_1855_MOESM1_ESM.docx]

**Appendix**

**19-item College of Optometrists in Vision Development Quality of Life (COVD-QOL) questionnaire**

| I.D. NUMBER： DATE： GRADE LEVEL：  Check the column which best represents the occurrence of each symptom | | | | | |
| --- | --- | --- | --- | --- | --- |
|  | Never | Seldom | Occasional | Frequently | Always |
| Headaches with near work | 0 | 1 | 2 | 3 | 4 |
| Words run together reading |  |  |  |  |  |
| Burn, itch, watery eyes |  |  |  |  |  |
| Skips/repeats lines reading |  |  |  |  |  |
| Head tilt/close one eye when reading |  |  |  |  |  |
| Difficulty copying from chalkboard |  |  |  |  |  |
| Avoids near work/reading |  |  |  |  |  |
| Omits small words when reading |  |  |  |  |  |
| Writes up/down hill |  |  |  |  |  |
| Misaligns digits/columns of numbers |  |  |  |  |  |
| Reading comprehension down |  |  |  |  |  |
| Holds reading too close |  |  |  |  |  |
| Trouble keeping attention on reading |  |  |  |  |  |
| Difficulty completing assignments on time |  |  |  |  |  |
| Always says “I can't” before trying |  |  |  |  |  |
| Clumsy, knocks things over |  |  |  |  |  |
| Does not use his/her time well |  |  |  |  |  |
| Loses belongings/things |  |  |  |  |  |
| Forgetful/poor memory |  |  |  |  |  |
| Other comments | | | | | |
